# Cross‐Saharan transport of water vapor via recycled cold pool outflows from moist convection

**DOI:** 10.1002/2016GL072108

**Published:** 2017-02-04

**Authors:** Tomasz M. Trzeciak, Luis Garcia‐Carreras, John H. Marsham

**Affiliations:** ^1^ School of Earth and EnvironmentUniversity of LeedsLeedsUK; ^2^ National Centre for Atmospheric ScienceLeedsUK

**Keywords:** water vapor transport, cold pool outflows, moist convection

## Abstract

Very sparse data have previously limited observational studies of meteorological processes in the Sahara. We present an observed case of convectively driven water vapor transport crossing the Sahara over 2.5 days in June 2012, from the Sahel in the south to the Atlas in the north. A daily cycle is observed, with deep convection in the evening generating moist cold pools that fed the next day's convection; the convection then generated new cold pools, providing a vertical recycling of moisture. Trajectories driven by analyses were able to capture the direction of the transport but not its full extent, particularly at night when cold pools are most active, and analyses missed much of the water content of cold pools. The results highlight the importance of cold pools for moisture transport, dust and clouds, and demonstrate the need to include these processes in models in order to improve the representation of Saharan atmosphere.

## Key Points

- Observed case of convectively driven water vapor transport across the Sahara over 2.5 days
- Cycle of convection generating cold pools which feed next day's convection, enhancing transport
- Analyses capture direction of transport but not its full extent due to missing cold pools

## 1 Introduction

During Northern Hemisphere summer a heat low develops over the Sahara, and the West African monsoon (WAM) brings the annual rains to the Sahel [*Sultan and Janicot*, 2003]. The Saharan Heat Low (SHL) is ventilated by cold air from its margins (including the WAM) with synoptic flow occurring at night, since it is inhibited by dry convection during the day [*Parker et al*., 2005; *Grams et al*., 2010]. The radiative balance of the SHL is affected by water vapor, clouds [*Stein et al*., 2011; *Evan et al*., 2014; *Marsham et al*., 2016], and dust [*Banks et al*., 2014], and the summertime Sahara represents the world's largest dust source [*Knippertz and Todd*, 2012]. Global models struggle to capture the WAM and variations in the SHL on daily, seasonal, and climate timescales [*Bock et al*., 2010; *Xue et al*., 2010], and known systematic biases in their representations of moist convection [*Marsham et al*., 2013b; *Birch et al*., 2014] and dust [*Johnson et al*., 2011; *Marsham et al*., 2011] are likely to contribute to this.

Up to 90% of the Sahel rains originate from organized mesoscale convective systems (MCSs) that tend to propagate westward or southwestward under the influence of the African Easterly Jet [*Mathon et al*., 2002]. Evaporation, melting, and sublimation of hydrometeors from the MCSs generate extensive cold pool outflows [*Provod et al*., 2015], which can spread over hundreds of kilometers and form the leading edge of the monsoon flow [*Flamant et al*., 2007]. In models with explicit convection, these cold pools make up a substantial fraction of the advective cooling by the monsoon, but cold pools are largely missing in model versions with parameterized convection [*Marsham et al*., 2013b], including meteorological analyses [*Sodemann et al*., 2015]. This lack of cold pools has been shown to significantly contribute to large‐scale systematic biases in temperature and humidity observed over the central Sahara [*Garcia‐Carreras et al*., 2013]. Cold pools also cause around 50% of summertime dust uplift in models with explicit convection that is missing in models with parameterized convection [*Marsham et al*., 2011; *Heinold et al*., 2013; *Pope et al*., 2016]. This large contribution of cold pools (“haboobs”) to summertime dust uplift is consistent with the observed link between the energy available to cold pools and the annual dust cycle [*Marsham et al*., 2008] and with 20 days of recent Fennec field campaign observations from the central Sahara [*Marsham et al*., 2013a; *Allen et al*., 2015]. These Fennec observations are from Bord Baji Mokhtar (BBM) in southern Algeria (21.4°N, 0.9°E), where in June cold pools and moisture were observed to arrive from the monsoon to the south, allowing the generation of local deep convection [*Marsham et al*., 2013a].

Deep convection over West Africa in summer is not restricted to the WAM, and, for example, storms also routinely form over the Atlas Mountains (~31°N), which can lead to southward propagating cold pools [*Knippertz et al*., 2007; *Emmel et al*., 2010] that weaken and moisten the SHL [*Redl et al*., 2016]. *Knippertz* [2003] and *Knippertz et al*. [2003] showed that especially during August and September moisture can be transported from the Sahel to the Sahara above the dry Saharan boundary layer (BL) at midlevels and that this can be associated with African Easterly Wave activity. In situ observations in the southern foothills of the Atlas Mountains, however, have found that of the 5 to 14 cold pool events measured every month in summer, 15% of cold pools arrive from the south [*Emmel et al*., 2010], potentially providing a different mechanism for cross‐Saharan moisture transport to that described by [*Knippertz*, 2003].

Here we describe a case study from June 2012 during the Fennec field campaign where we find that cold pools formed a key component of the moisture transport from the Sahel to feed convective storms over the Atlas: moisture arrived in the central Sahara in cold pools from MCSs in the Sahel and then was transported across the Sahara over 2.5 days to the Atlas Mountains. During each day of the trans‐Saharan transport, moisture from cold pools (generated by the previous night's convection) fed deep convection that developed in the afternoon and evening, which in turn generated a new cold pool. This case is in contrast with [*Knippertz*, 2003] where the northward moisture transport occurred primarily at mid levels. Section 2 describes the data sets and methods used, section 3 discusses the results of the test case, and section 4 summarizes and discusses the implications of the results in more detail.

## 2 Method

**Figure 1:**
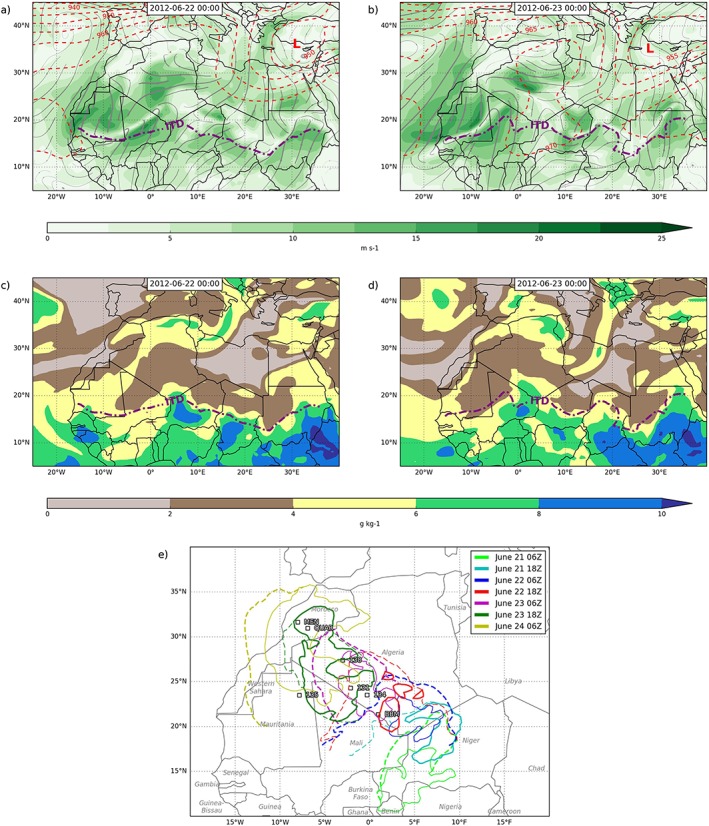
Synoptic state at 00 UTC on (a and c) 22 June and (b and d) 23 June. Figures 1a and 1b show 925 hPa wind speed (colors), streamlines (grey), 300 hPa geopotential height (dashed red contours, gpdm), and Intertropical Discontinuity (ITD) defined by the 14°C isotherm of 2 m dew point temperature (dash‐dotted purple line); Figures 1c and 1d show 925 hPa water vapor mixing ratio (colors) and ITD as above. (e) Schematic of daily locations of deep convective cloud (solid lines) and cold pools (dashed lines), at 06 UTC (thin lines) and 18 UTC (thick lines).

The synoptic conditions present during the case study are described using the ERA‐Interim global reanalysis produced by the European Centre for Medium‐Range Weather Forecasts [*Dee et al*., 2011], with data at 0.25° and 6‐hourly resolutions (Figure 1). ERA‐Interim data are also compared to ground station data (which were not assimilated) and radiosondes from the central Sahara (which were assimilated).

**Figure 2:**
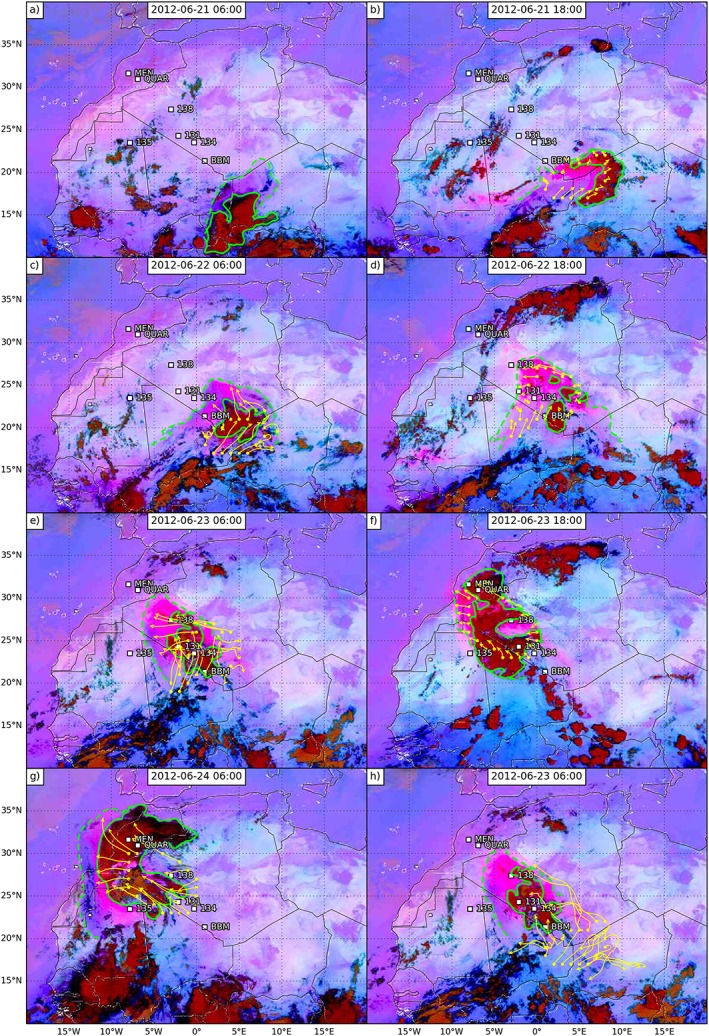
The 12‐hourly SEVIRI images from (a–g) 06 UTC on 21 June to 06 UTC on 24 June. Dark red colors represent cold cloud, while bright pinks show dust, which can be used to track the movement of dusty cold pools. Overlaid trajectories are initialized each day at 12 UTC at 1 km above ground level over uplifted dust regions, with dots showing the start points and arrowheads showing the end points. (h) Same as Figure 2e but showing trajectories initialised at 12 UTC on 21 June and run continuously for 2.5 days.

Imagery derived from the Spinning Enhanced Visible and Infrared Imager (SEVIRI) on board the Meteosat Second Generation geostationary satellites are used to identify dust and cloud features (shown in pink and dark red, respectively) [*Lensky and Rosenfeld*, 2008; *Brindley et al*., 2012]. These data are available at 15 min and ~3 km resolution (at nadir) and so are particularly suited for tracking fast‐moving features such as cold pools and convective storms (Figure 2). Cold pools in particular can be identified in the dust channels, due to their generation of intense dust uplift (pink in the satellite imagery) in a distinctive pattern with a spreading area of dust with a sharp leading edge emanating from deep convection (red/black in the imagery). Similar use of satellite imagery in this way, with the support of surface data, can be found, for example, in *Marsham et al*. [2013a].

To quantify synoptic transport in global atmospheric models and determine their ability to represent the transport mechanisms presented in this paper, trajectories were run using the Hybrid Single‐Particle Lagrangian Integrated Trajectory (HYSPLIT) Model [*Stein et al*., 2015] driven by the National Centers for Environmental Prediction Global Data Assimilation System model data at 3‐hourly and 1° resolution (http://ready.arl.noaa.gov/gdas1.php). The trajectories were reinitialized at 12 UTC each day 1 km above ground level nearby the edges of dusty cold pool regions identified visually with SEVIRI data for a grid of points 1° apart and then run forward for 18 h (Figure 2). A height of 1 km was used as this is typically within a cold pool outflow [e.g., *Garcia‐Carreras et al*., 2013], and results were only weakly sensitive to the height used (not shown). A set of trajectories were also initialized at 12 UTC on 21 June and run forward for 3 days to determine the model's ability to represent the transport throughout the case study (Figure 2h).

**Figure 3:**
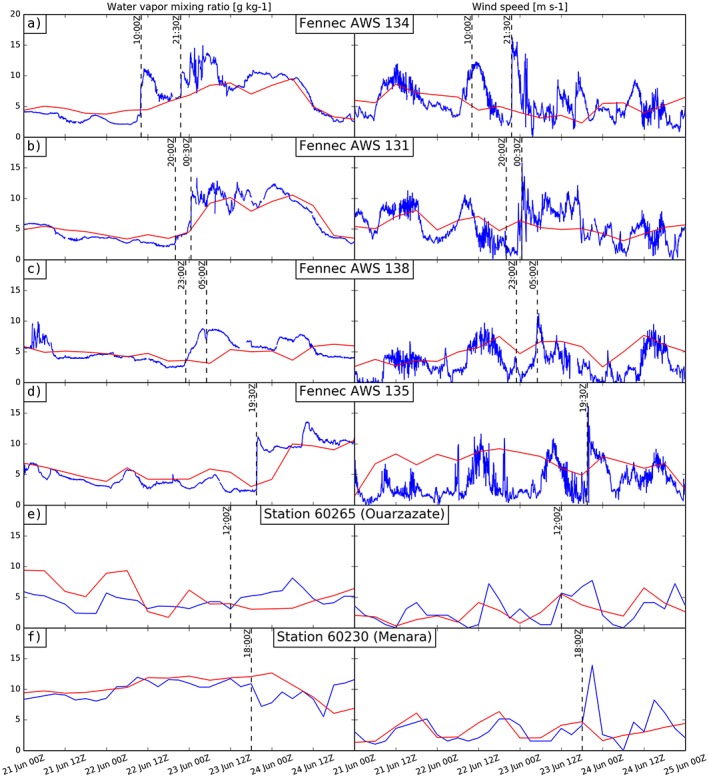
Water vapor mixing ratio (left column) and wind speed (right column) at (a–d) four automatic weather stations and (e and f) two synoptic stations in the central and northern Sahara (blue lines, see Figure 2 for locations), and ERA‐Interim values at the same locations (red lines). Vertical dashed lines show the time of cold pool crossings over the stations.

**Figure 4:**
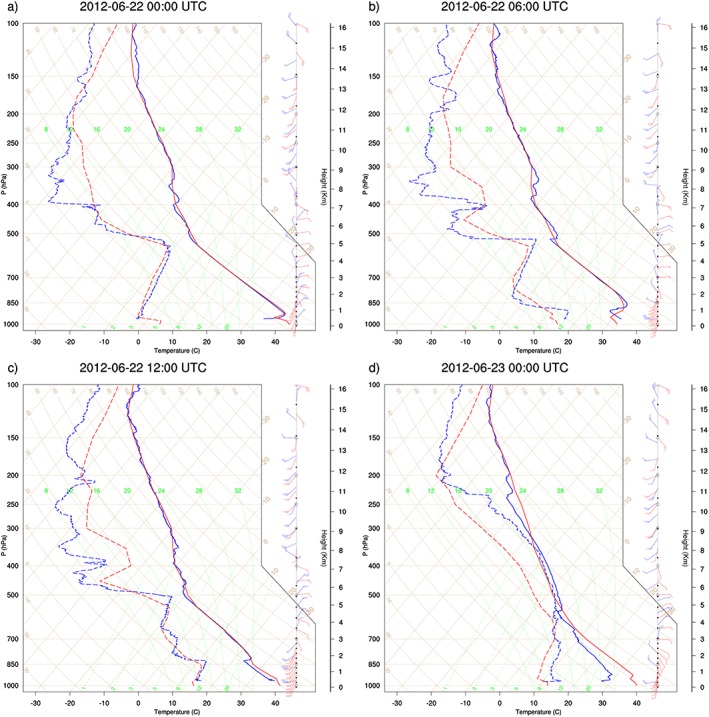
Tephigrams from BBM radiosonde profiles (blue) and ERA‐Interim profiles (red) on 22 June at (a) 00 UTC, (b) 06 UTC, (c) 12 UTC, and (d) 18 UTC.

Surface weather station data from the Fennec project network [*Hobby et al*., 2013] were used to characterize cold pool properties as they propagated across the Sahara (Figure 3). The data comprise 2 m absolute humidity and wind speed measurements from four automatic weather stations in Algeria (131, 134, and 138) and Mauritania (135). Vaisala RS92 radiosondes from Fennec supersite 1 at Bordj Baji Mokhtar (BBM) [*Marsham et al*., 2013a] were also used (Figure 4), as well as data from two synoptic stations in Morocco (OUAR and MEN) (Figure 3). All locations are shown in Figures 1e and 2.

## 3 Results

The case study covers the period from the evening of 21 to the morning of 24 June 2012. Figure 1 shows the synoptic situation for the case study with ERA‐Interim reanalysis data on 22 (Figures 1a and 1c) and 23 June (Figures 1b and 1d). Previous case studies have highlighted the role of midlevel moisture transport from the tropics in generating convection over the Atlas in “tropical‐extratropical interactions (TEIs)” [*Knippertz*, 2003]. The synoptic situation shown in Figure 1, however, differs from the schematic of TEIs shown in *Knippertz* [2003, Figure 1]. In both cases there is an upper level anticyclone over northern Algeria (dashed red lines, Figures 1a and 1b), but the TEIs described in *Knippertz* [2003] have a prominent upper level trough west of Morocco and Mauritania that is absent in Figure 1. Instead, the change in wind field from Figure 1a to Figure 1b shows moisture transport from the Sahel toward the Moroccan Atlas occurs at low levels (see *Barthe et al*. [2010] for a 2006 monsoon surge case). On 22 June there is a monsoon surge over the border triple point of Algeria‐Niger and Mali, with southwesterly winds at 925 hPa extending as far the Intertropical Discontinuity (as defined using the minimum in wind speed, Figure 1a) which reaches 24 N in southern Algeria (with the Intertropical Discontinuity (ITD), as defined by the 14°C isotherm of 2 m dew point temperature just to the south). By 23 June the ITD has moved northwest toward northern Mali and Mauritania. Figures 1c and 1d show the low‐level moisture transport associated with the monsoon surge, which spreads from the Sahel deep into the Sahara. The humidity transport extends up to the mid levels (700 hPa, not shown) but is tied to the monsoon surge shown in Figures 1c and 1d. In the rest of the results we will first examine the role of moist convection and cold pools in this transport using satellite imagery, followed by in situ measurements of cold pool properties and their impact on the vertical atmospheric profile.

Figures 2a–2g shows 12‐hourly SEVIRI imagery from 06 UTC on 21 June 2012 through the next 2.5 days to 06 UTC on 24 June 2012. We encourage the reader to view the animation of the 15 min resolution satellite imagery which is particularly helpful in illustrating the evolution of the case study. Figure 1e is a schematic summarizing the events of the entire case study as determined from the satellite imagery in Figure 2, showing the daily evolution of the cold pools and deep convection. Figure S1 in the supporting information is like Figure 2 but for times when cold pools cross specific observation stations. In these images, regions of deep cloud are shown as dark reds, while dust is bright pink. Overlaid trajectories are reinitialized at 12 UTC each day over uplifted dust regions.

Figure 2a shows an MCS over southwest Niger (15°N, 5°E) which was initiated over southeast Niger on the afternoon of 20 June (not shown). A cold pool can be seen propagating northwestward close to the Niger‐Algeria‐Mali triple point as a region of enhanced dust. By 18 UTC the MCS has continued to propagate west reaching northern Burkina Faso (13°N, 0°E, Figure 2b), while the cold pool outflow has become more extensive, leading to a dusty region centered at 20°N, 5°E in Figure 2b. Cumulonimbus cells were initiated on the edge of this cold pool at 15 UTC, east of the Mali‐Algeria‐Niger border triple point (visible at 18 UTC in Figure 2b). The new cells produced their own outflows, which merged with the preexisting outflow and can be seen spreading northwards in Figures 2b and 2c, crossing BBM at around 01 UTC on 22 June (Figure S1a).

During 22 June the aged cold pool was advected northwest in a distinctive hook shape, which is still visible as a distinct dusty (bright pink) air mass in Figure 2d. New cumulonimbus cells are initiated over the old cold pool east of BBM and over the Hoggar Mountains at 15 UTC (20°N, 4°E in Figure 2d) and by 00 UTC on 23 June BBM is covered by moist convection. These new storms generate new cold pools traveling northwestward, eventually merging with the aged cold pool from the previous night.

During the morning of 23 June the convection over BBM begins to dissipate, while the pink cold pool outflow continues to move northwestward reaching the very north of Mali and northeast of Mauritania (Figure 2e), and extending farther into northern Mauritania and south Morocco throughout the day (Figure 2f). From midday convection redeveloped over the old cold pool northwest of BBM in northern Mali, generating an extensive westward propagating cold pool. Around midday of 23 June new cells also developed over the old cold pool south and east of Ouarzazate with fresh cold pools clear in SEVIRI imagery from 14 UTC. Convection then developed along the Atlas Mountains, north of Ouarzazate and close to the northern edge of the cold pool, from 16 UTC on 23 June (seen at 18 UTC in Figure 2f). Overnight the dust and convection moved westward over north Mali and extended over the Moroccan coast (Figure 2g).

The trajectories show that the model is able to reproduce the direction of the synoptic transport during the day but fails to capture the transport from propagating cold pools, which is unsurprising as the analyses driving the trajectories are not expected to capture the dynamics of the fresh cold pool outflows (green lines, Figure 2). For example, the trajectories initialized at 12 UTC on 21 June and run for 18 h capture the hooked shape of the cold pool but not its full extent (Figure 2c), while the trajectories on the following day do capture the advection of the aged cold pool between 12 and 18 UTC (Figure 2d) presumably because it is no longer acting as a fresh density current. Similarly, the trajectories at 06 UTC on both 23 and 24 June (Figures 2e and 2g) do not capture the full extent of the active cold pool, while the daytime trajectories (18 UTC, Figure 2f) are much better at capturing the northwestward movement of the entire aged cold pool, although it misses the impact of fresh cold pools spreading southwest into north Mali and northeast Mauritania.

Figure 2h shows trajectories initiated from inside the cold pool at midday on 21 June and run continuously until 06 UTC on 23 June. The trajectories capture the northwestward movement of the monsoon surge but only extend as far as western central Algeria, whereas the real moist dusty air by this time had almost reached the Atlas Mountains. It is therefore clear that the cold pools not only provide a vertical recycling mechanism for moisture, where evaporated overnight rainfall feeds the next evening's convection, but are an integral component of the cross‐Saharan moisture transport. The cold pools therefore generate northwestward advection of moisture that is missing in the trajectories.

Figure 3 shows time series of water vapor mixing ratio (WVMR) and wind speed at four Automatic Weather Stations (AWSs) and two surface SYNOP stations, with dashed lines indicating the passage of cold pools (AWS locations in Figures 1e and 2, with corresponding imagery in supporting information Figure 1). The in situ data confirm that the high‐dust features in the satellite imagery correspond to cold pools; as in all cases their arrival coincides with distinct changes in both specific humidity and wind speed. Recently generated cold pools are associated with increases in humidity from between 2 and 6 g kg^−1^ to 11 to 13 g kg^−1^ and wind gusts of 13 to 15 m s^−1^ (2130 UTC on 22 June at AWS134, 0030 UTC on 23 June at AWS 131, and 1930 UTC on 23 June at AWS 135). The aging cold pool that arrived at AWS134 at 10 UTC on 22 June is notable in that winds increase before the specific humidity, suggesting that the cold pool was located above the nocturnal boundary layer and moisture and momentum were mixed downward from above as the dry convective BL developed (as shown in modeling of a different case by *Heinold et al*. [2013]). By the evening, this aged cold pool produces a much smaller, but detectable, increase in specific humidity of 1 g kg^−1^ at AWS131 at 20 UTC, with no wind speed increase. This cold pool arrives at AWS138 at 23 UTC, and here the station humidity continues to increase overnight as more moisture is advected, with wind increasing by 3 m s^−1^. This cold pool is reinforced by convection developing over it to the south and at 05 UTC on 23 June a new cold pool gives a large increase in wind speeds of 10 m s^−1^, but a short‐lived reduction in humidity from 9 to 7 g kg^−1^ as it displaces the preexisting moist cold pool air.

The surface SYNOP stations only provide 6‐hourly data, so the exact timing of the passage of cold pools cannot be identified. However, the passage of a cold pool in the afternoon of 23 June at Ouarzazate is accompanied by an increase in specific humidity from 3 g kg^−1^ at 12 UTC to 8 g kg^−1^ by 06 UTC on 24 June and a persistence of high winds into the night on 23 June unlike the preceding days. At Menara, on the other hand, this cold pool leads to a dramatic peak in wind speed of 13 m s^−1^, but a drying since the moist (8 g kg^−1^) cold pool was drier than the Atlantic‐influenced air ahead of the cold pool on the northern side of the Atlas (12 g kg^−1^), compared with the 4 g kg^−1^ that preceded the cold pool at Ouarzazate on the south side of the Atlas (Figures 3e and 3f).

BBM radiosondes on 22 June provide an example of the vertical changes induced by the cold pool passage. Between 00 UTC and 06 UTC, before and after the passage of the cold pool (Figures 3a and 3b), there is a low‐level moistening from 3 to 12 g kg^−1^ and a cooling of around 5 K associated with the cold pool, consistent with the AWS results. The increased humidity increases convective available potential energy (CAPE) from 0 to 346 J kg^−1^ between 00 and 06 UTC, with daytime heating allowing CAPE to reach 1118 J kg^−1^ by 12 UTC. BBM radiosondes show a deepening of the low‐level moist layer between 06 and 12 UTC, consistent with upward mixing of moisture from the cold pool (Figures 3b and 3c), allowing deep convection by 18 UTC (Figure 2d and moist adiabatic profile in Figure 3d).

ERA‐Interim, which did not assimilate the AWS data, either misses the cold pool events described above or captures the increases in humidity over 6 h late (red lines, Figure 3). This is consistent with the trajectories which capture the correct general transport but underestimate its full extent. *Sodemann et al*. [2015] similarly found that reanalysis data were insufficient to fully describe dust transport in the region due to missing deep convective activity and cold pools. At AWS 134 and 131 the analysis misses the first, aged cold pool (10 UTC At AWS134, 20 UTC at AWS131), while the humidity increase due to the arrival of the second cold pool (2130 at AWS134 and 0030 at AWS131) is ~6 h late, and particularly for AWS 134, considerably underestimated in magnitude. At both stations, wind increases associated to these cold pools are completely missing. Similarly, at AWS135 the humidity increase associated with the cold pool passage is 12 h late, with only a very minor increase in wind speeds (2 m s^−1^, compared to 15 m s^−1^ in observations, Figure 4d). At AWS 138 the cold pools appear to be completely missing, with only a minor increase in WVMR after the second cold pool (Figure 4c). At Ouarzazate and Menara the cold pools are also missing, with opposite trends in humidity compared to observations at the cold pool passage times (dashed lines in Figures 4e and 4f). Even at BBM, where Fennec radiosondes were assimilated, ERA‐Interim misses the highest WVMRs from the cold pool at 06 UTC on 22 June (Figure 4b), and although it captures the subsequent evolution (Figure 4c), it again misses details of the next night's convection and cold pools (Figure 4d).

In summary, Figure 2 shows that moist convection was initiated on the edge of a cold pool generated by a Sahelian MCS on the evening of the 21 June on the southern side of the Sahara. This convection formed its own outflows. Over the next 2 days fresh moist convection was initiated each evening on the cold pools from the preceding day's convection. The cold pools and convection moved northwestward across the Sahara to reach and cross the Atlas, triggering convection there on the evening of the 23 June. In situ data show that cold pools gave sudden increases in moisture (similar to premonsoon cold pools in the Sahel [*Provod et al*., 2015]) when they arrived at stations in the Sahara, allowing for high CAPE: in contrast, on the Atlantic side of the Atlas they gave a drying due to a preexisting moist boundary layer. Over the Sahara, the cold pools were warmed during the day, reducing inhibition allowing subsequent convection. The cold pools gave strong surface winds, lofting dust making them visible in SEVIRI imagery. Trajectories captured much of the northwest progress of the cold pools, which were embedded in a northwest surge of the monsoon (Figure 1). As expected, however, they did not capture the extent of the freshly generated cold pools, which are also largely missing in ERA‐Interim.

## 4 Conclusions

We have described a case study of water vapor transport from the Sahel over the Sahara to the Moroccan Atlas over 2.5 days. The transport was driven by a daily cycle of deep convective storms in the evening which generated cold pools that fed the next day's convection; the convection then generated new cold pools, providing a vertical recycling of moisture. This northwestward transport of moisture occurs due to the synoptic conditions but is augmented by the cold pools acting as density currents and transporting moisture. Trajectories therefore capture the direction of the transport but not its full extent, particularly at night when cold pools are most active.

The conclusion that cold pools are a key component of moisture transport is consistent with past modeling studies [*Marsham et al*., 2013b] and statistics of model errors from the Fennec supersite in the central Sahara [*Garcia‐Carreras et al*., 2013], but this case study demonstrates the mechanisms. The case shows the importance of low‐level cold pool‐enhanced moisture transport from the Sahel across the Sahara to feed convection over the Atlas, which is unlike the midlevel transport shown in *Knippertz* [2003]. Although it was the clearest case of such transport noted during the Fennec period, the fact that 15% of summertime Atlas cold pools arrive from the south [*Emmel et al*., 2010] suggests that it is not that unusual. Around 19 cold pools were observed at BBM in June 2011 during 24 days with regular cloud cover [*Marsham et al*., 2013a], and SEVIRI imagery from the Sahara suggests that the daily recycling of water through cold pools and subsequent convection is extremely common. While cold pools are known to trigger storms through lifting in many regions, they play a distinct role in the Sahara (and other similarly arid regions), as here they are much moister than their environment, and once rewarmed they provide the source air for new storms, whereas in many regions they are drier, since they consist of descending air (e.g., *Provod et al*. [2015] for the Sahel after the monsoon onset or *Bryan and Parker* [2010] in the U.S.). Cold pools are generally missing in both models with parameterized convection [*Marsham et al*., 2011; *Garcia‐Carreras et al*., 2013] and even meteorological analyses over the Sahara [*Knippertz et al*., 2007]. It is clear that the representation of cold pools must be addressed if models are to capture the water vapor transport and convection over the Sahara, which is key to its radiative balance [*Evan et al*., 2014; *Marsham et al*., 2016], dust uplift [*Marsham et al*., 2011, 2013a], and high‐impact weather.

## Supporting information

Supporting Information S1

Movie S1
