# Supplementary material for: Cross‐Saharan transport of water vapor via recycled cold pool outflows from moist convection
Source: Geophys Res Lett. 2017 Feb 4;44(3):1554–63. doi: 10.1002/2016GL072108 (PMC5347875; doi:10.1002/2016GL072108)
Supplement: Supplementary file 1 — Supporting Information S1 [file GRL-44-1554-s001.pdf]

AGU PUBLICATIONS

Geophysical Research Letters

Supporting Information for

Cross Saharan transport of water vapour via recycled cold-pool outflows from moist convection

Tomasz Trzeciak[1], John H Marsham[1,2], Luis Garcia Carreras[1]

[1]School of Earth and Environment, University of Leeds, UK

[2]National Centre for Atmospheric Science (NCAS), UK

Contents of this file

Figure S1

Additional Supporting Information (Files uploaded separately)

Captions for Movie S1

Introduction

Imagery derived from the Spinning Enhanced Visible and Infrared Imager (SEVIRI) onboard the Meteosat Second Generation geostationary satellites are used to identify dust and cloud features (shown in pink and dark red respectively), see the method section in the main text. These data are available at 15-minute and ~3 km resolution (at nadir), and so are particularly suited for tracking fast-moving features such as cold pools and convective storms. Cold pools in particular can be identified in the dust channels, due to their generation of high dust uplift.

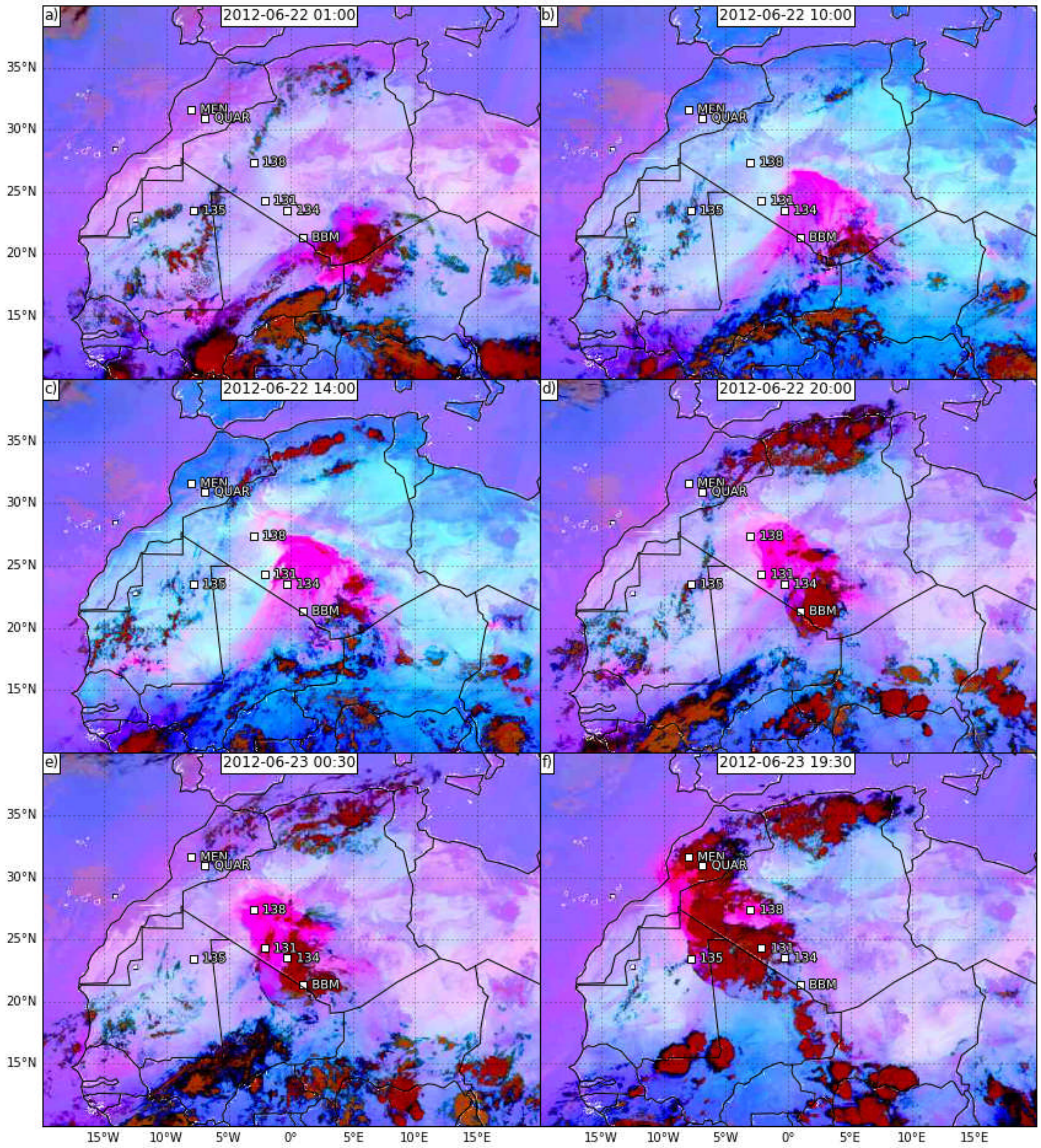

Figure S1. SEVIRI images at times when cold pools cross specific observation stations (as shown by the vertical dashed lines in Figure 4).

Movie S1. Animation of SEVIRI images from the period of the case-study, showing the cycle of deep moist convection forming cold pool outflows, which trigger further convection the next day.
