# Supplementary figures and images for: Cross‐Saharan transport of water vapor via recycled cold pool outflows from moist convection
Source: Geophys Res Lett. 2017 Feb 4;44(3):1554–63. doi: 10.1002/2016GL072108 (PMC5347875; doi:10.1002/2016GL072108)

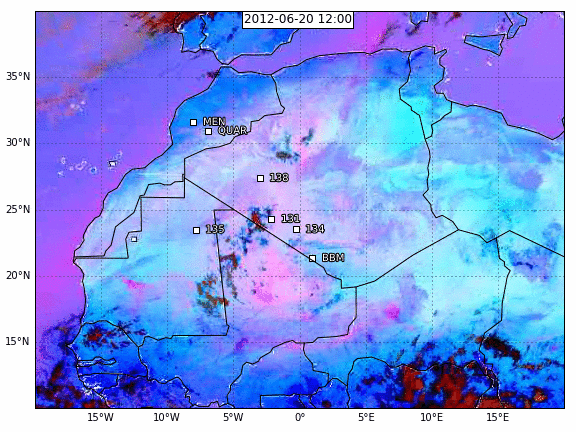

Supplement: Supplementary file 2 — Movie S1 [file GRL-44-1554-s002.gif]
